# Supplementary material for: A Novel Non-Invasive Murine Model of Neonatal Hypoxic-Ischemic Encephalopathy Demonstrates Developmental Delay and Motor Deficits with Activation of Inflammatory Pathways in Monocytes
Source: Cells. 2024 Sep 14;13(18):1551. doi: 10.3390/cells13181551 (PMC11429599; doi:10.3390/cells13181551)
Supplement: Supplementary file 1 [file cells-13-01551-s001.zip › Supplemental Figures.pdf]

Supplemental Figures

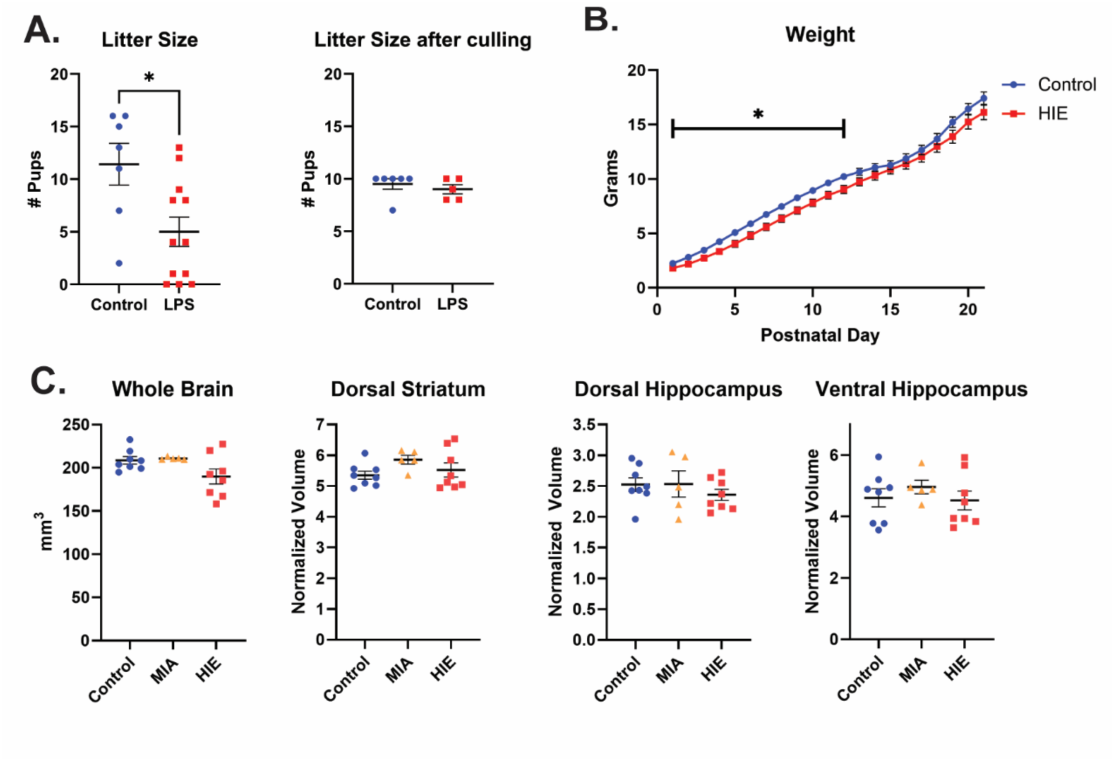

Figure S1. Additional neonatal data (A) LPS administration to pregnant dams significantly decreased the number of live pups on P0, the day of birth ($t(17) = 2.714$, $p = 0.0147$, unpaired t-test). To control for litter size effects on developmental behaviors due to maternal care, litters were culled to a maximum of 10 pups, and litters of 6 pups or smaller were excluded from behavioral analysis. This eliminated the litter size difference between groups ($F(1.54,29.23) = 789.5$, $p < 0.0001$, two-way ANOVA). **(B)** Beginning on P0, LPS pups weighed less than controls, and this difference persisted until P13 ($F(1,19) = 4.940$, $p = 0.0386$, two-way ANOVA). From P13 through P21, the time of weaning, there was no difference in weight between the groups. **(C)** There was a trend towards significance in a decrease of the whole brain volume that appears to be driven by the two-hit HIE animals ($F(2,18) = 3.270$, $p = 0.0615$, one-way ANOVA). There were no differences between groups in brain region volumes measured by *ex vivo* MRI on P7. (n = 8 control; 5 MIA; 8 HIE).

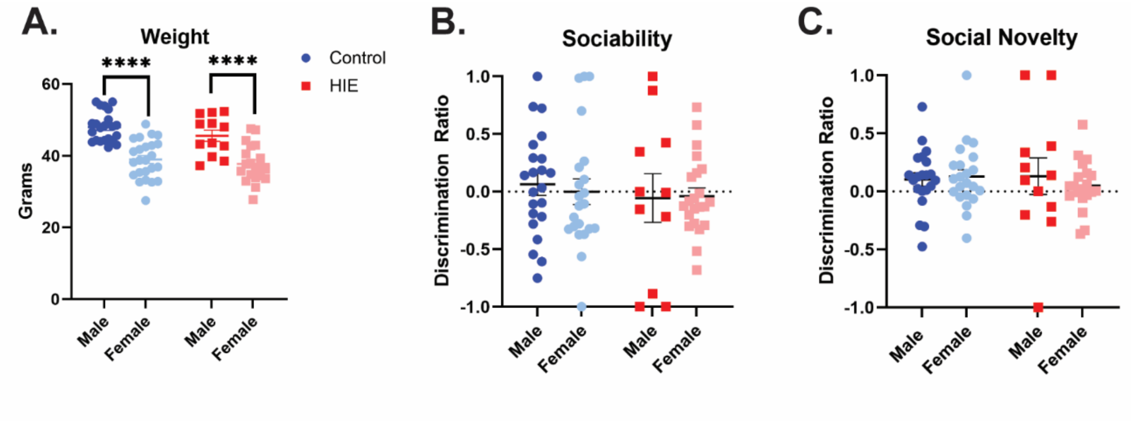

Figure S2. Additional adult data. (A) In adulthood (P60) females weighed significantly less than males ($F(1,74) = 51.28$, $p < 0.0001$, two-way ANOVA) There was no difference between HIE and control animals ($F(1,74) = 2.396$, $p = 0.126$, two-way ANOVA). **(B)** There were no differences in discrimination ratio in three-chamber sociability (F(1,73, = 0.7268, $p = 0.498$, two-way ANOVA) or **(C)** three-chamber social novelty ($F(1, 71) = 0.1169$, $p = 0.7444$, two-way ANOVA). Some animals were excluded from analysis due to issues with video recordings.

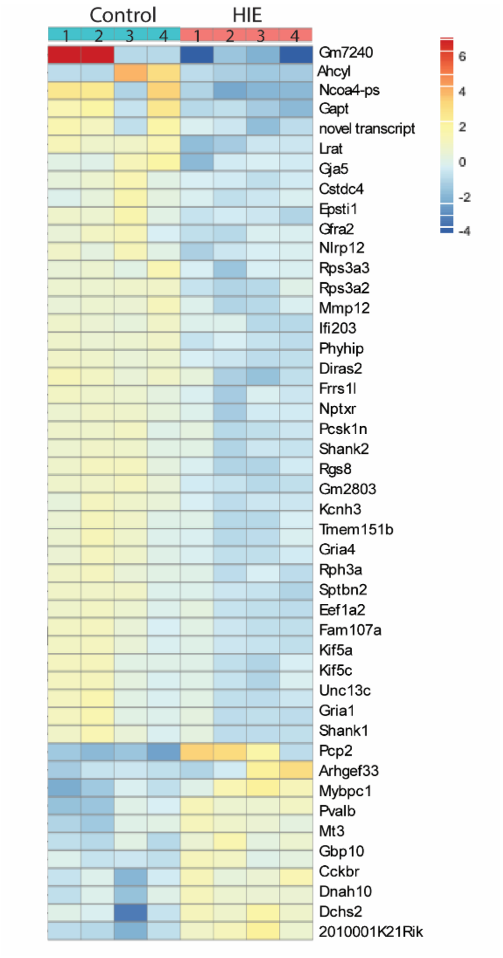

Figure S3. Microglia bulk RNAseq P14. Top upregulated and downregulated genes identified by both DESeq2 and edgeR with an FDR adjusted p-value < .05 within CD11b+ cells extracted from whole brain eight days following a second hit of hypoxia.

Table S1: Microglia Downregulated GSEA Analysis P7

Hallmark Gene Set	ES	NES	FDRq-val	FWER p-val	Rank at Max
Epithelial Mesenchymal Transition	-0.59	-2.10	< 0.001	< 0.001	4158
Myogenesis	-0.48	-1.71	0.003	0.015	4705
Estrogen Response Early	-0.43	-1.54	0.015	0.131	5676
Estrogen Response Late	-0.42	-1.48	0.026	0.560	6006

ES: Enrichment Score; NES: Normalized Enrichment Score; FDR: False Discovery Rate; FWR: Familywise-error Rate.

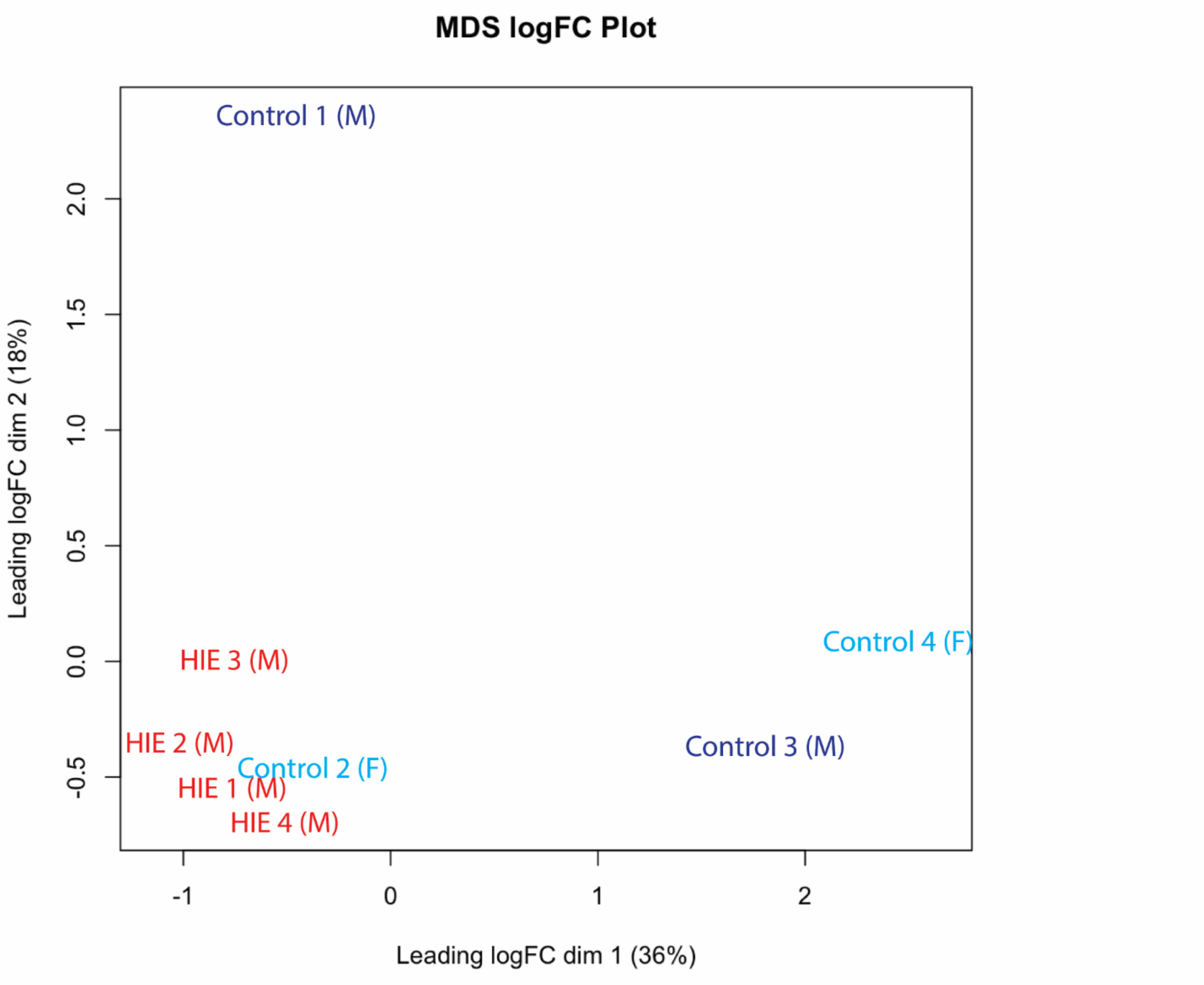

Figure S4. Bulk RNAseq data (P7) multidimensional scaling plot. PCA representation of the stratification of bulk RNAseq data based on HIE group and sex.

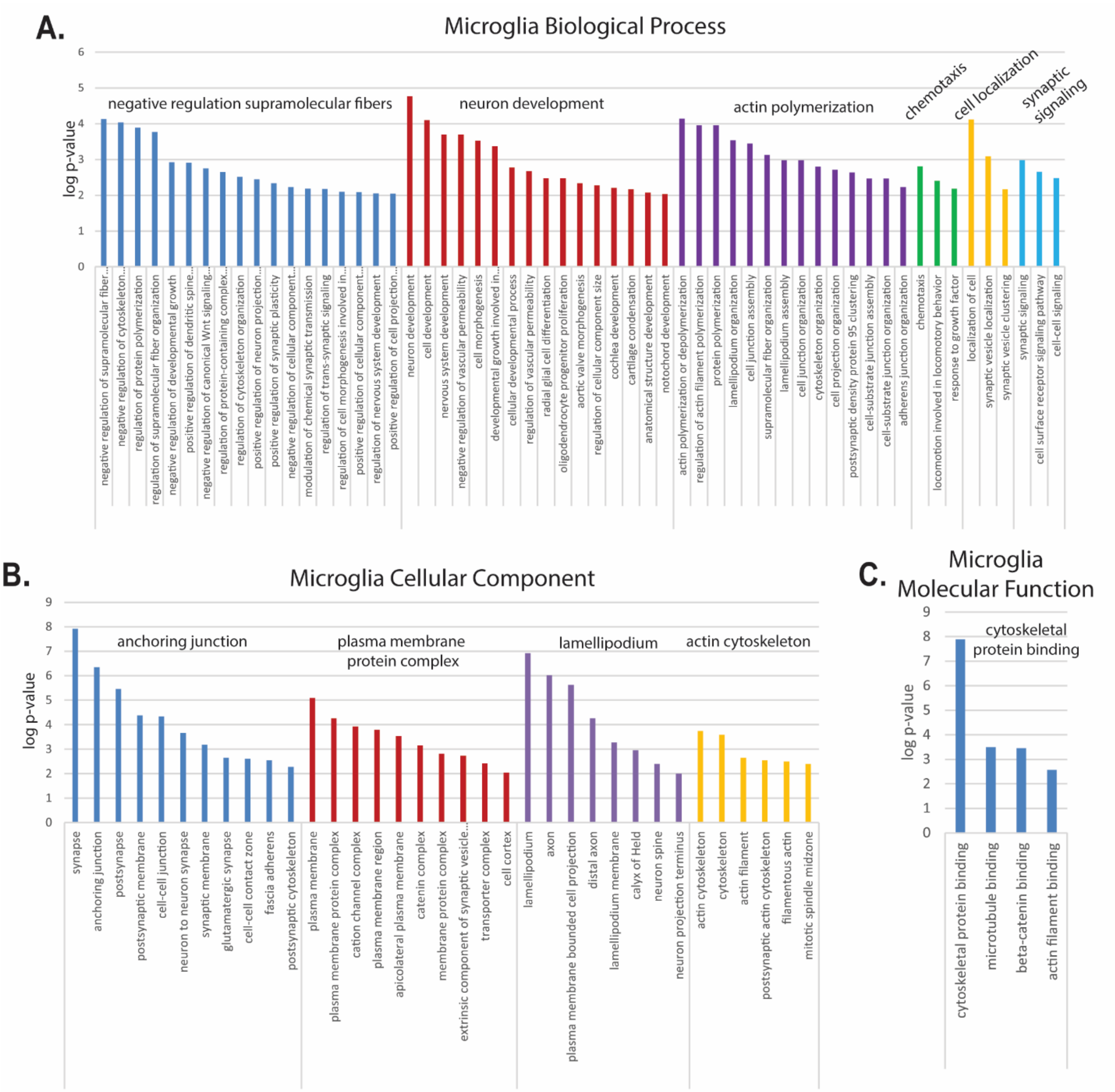

Figure S5. Microglia ReviGO Analysis. (A) Microglia biological process ReviGO groups. **(B)** Microglia cellular component ReviGO groups. **(C)** Microglia molecular function ReviGO groups.

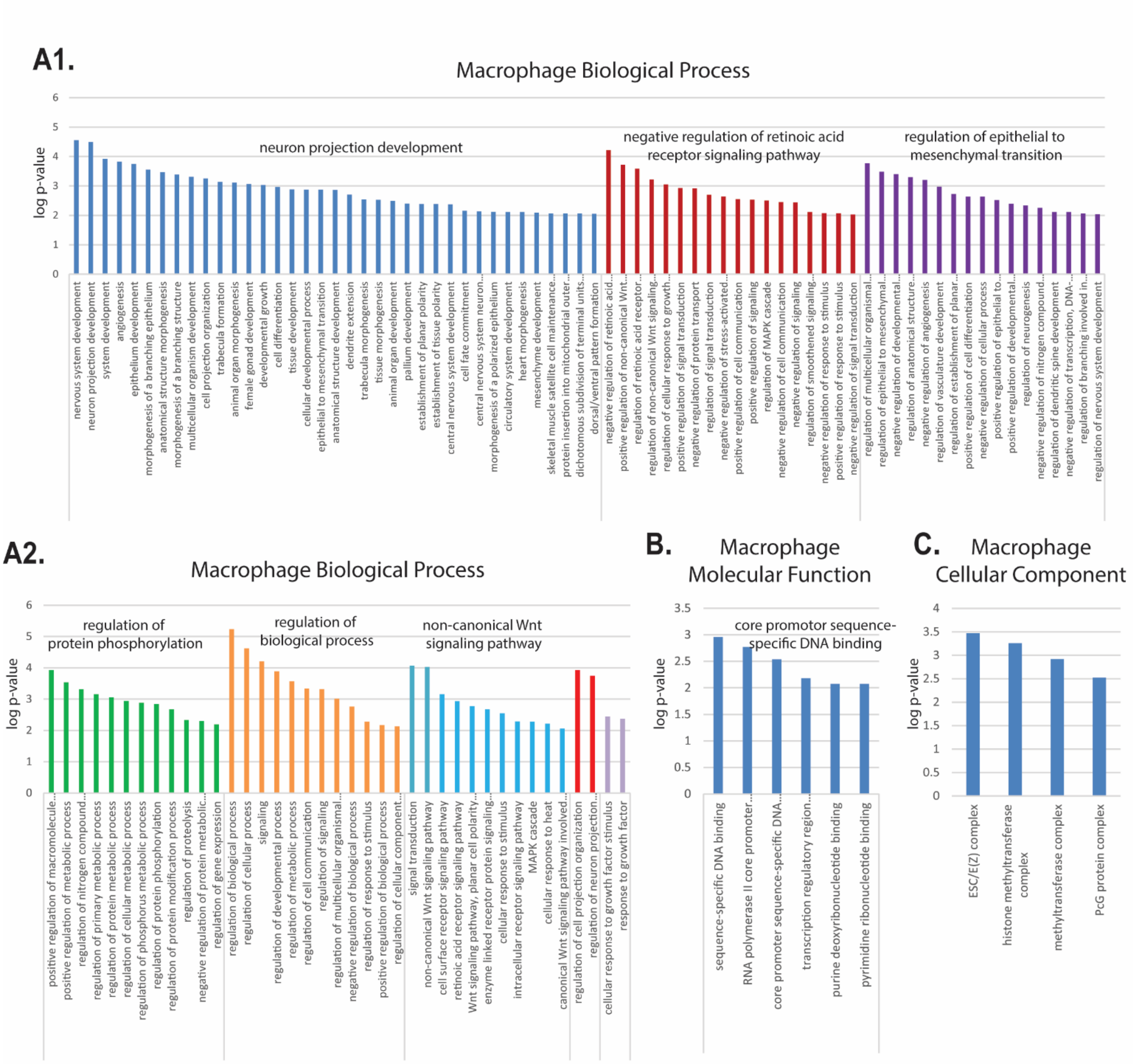

Figure S6. Macrophage ReviGO Analysis. (A) Macrophage biological process ReviGO groups. **(B)** Macrophage cellular component ReviGO groups. **(C)** Macrophage molecular function ReviGO groups.
